# Retrospective Study of the Epidemiological–Clinical Characteristics of Burns Treated in a Hospital Emergency Service (2018–2022)

**DOI:** 10.3390/nursrep14030148

**Published:** 2024-08-14

**Authors:** María Alcalá-Cerrillo, Josefa González-Sánchez, Jerónimo J. González-Bernal, Mirian Santamaría-Peláez, Jessica Fernández-Solana, Sara M. Sánchez Gómez, Ana Gómez-Martín

**Affiliations:** 1 Social Impact and Innovation in Health, Faculty of Nursing and Occupational Therapy, University of Extremadura, Avda. de la Universidad s/n, 10003 Cáceres, Spain; malcala@unex.es; 2 Health Science Department, Faculty of Health Science, University of Burgos, Paseo de los Comendadores s/n, 09001 Burgos, Spain; mjgonzalez@ubu.es (J.G.-S.); jejavier@ubu.es (J.J.G.-B.); jfsolana@ubu.es (J.F.-S.); 3 Nursing Department, Faculty of Nursing and Occupational Therapy, University of Extremadura, Avda. de la Universidad s/n, 10003 Cáceres, Spain; smsanchezg02@educarex.es (S.M.S.G.); anagomez@unex.es (A.G.-M.); 4 Extremadura Institute of Biosanitary Research (Instituto de Investigación Biosanitaria de Extremadura—INUBE), 1003 Cáceres, Spain

**Keywords:** burns, hospital emergency service, ICD-10 classification

## Abstract

Background: Burns are a common and severe medical emergency requiring immediate specialized care to minimize damage and prevent complications. Burn severity depends on depth, extent, and location, with more complex care needed for burns on critical areas or extensive burns. Nursing is essential in burn management, providing immediate care, adapting treatments, managing pain, preventing infections, and offering emotional support for recovery. The study aims to analyse the epidemiological and clinical characteristics of burns treated at the Hospital Emergency Department of the Hospital Complex of Cáceres (Spain) from January 2018 to December 2022. It looks at factors like gender, age, hospital stay duration, emergency type (paediatric or adult), main diagnosis, skin thickness, burn degree, affected body areas, percentage of body surface area burned, and treatment types. It also investigates how treatment varies by gender, age, skin thickness, and burn severity. The relevance of this research lies in the fact that periodic epidemiological studies are essential to monitor changes in diseases, evaluate the effectiveness of interventions, detect outbreaks quickly, update knowledge on risk factors, and guide health policy decisions. This ensures an adapted and effective response to the needs of the population. Methods: Retrospective, observational study that analysed burn cases treated at the Hospital Complex of Cáceres (Spain) 2018–2022. Inclusion criteria were based on ICD-10 codes for burns, excluding severe cases not treated in this service. Data were analysed using descriptive statistics, Student’s *t*-tests, Chi-square tests, and ANOVA. Results: 220 patients surveyed, with a mean age of 47 years and 60.9% male. Most burns (95.5%) affected the external body surface, with a mean hospital stay of 7.86 days. Medical treatment was provided to 75.5% of patients, and 24.5% required surgical intervention. Significant differences in treatment procedures were observed according to age, skin thickness, and burn degree. Older patients had more procedures and longer hospital stays. Excision and transfer procedures were more common in full-thickness and severe burns. Conclusions: The findings align with previous research on burn demographics and treatment approaches. Treatment differences by age and burn severity highlight the need for tailored interventions. The study underscores the importance of comprehensive burn management, including psychological support for improved long-term outcomes. Further research could explore the impact of socio-economic factors on burn incidence and treatment. This study was not registered.

## 1. Introduction

Burns represent one of the most common and severe medical emergencies seen in emergency departments. They are traumatic injuries resulting from exposure to various sources of heat, electricity, chemicals or radiation, and their treatment involves immediate and specialized care to minimize damage and prevent complications [1]. Burns can be classified according to their depth into superficial, partial-thickness and full-thickness burns. Superficial burns affect only the epidermis, while partial-thickness burns affect the dermis and full-thickness burns penetrate to underlying tissues such as muscle and bone [2,3]. In addition, the extent of burns is commonly measured using the rule of nines or the Lund and Browder chart, which allows for estimating the percentage of body surface area affected, which is crucial for determining the severity of the injury and planning appropriate treatment [2,4].

The depth of a burn directly affects both its prognosis and the required treatment. Superficial burns, which only involve the epidermis, generally need only basic care and heal quickly. In contrast, partial-thickness and full-thickness burns, which penetrate deeper into the dermis and underlying tissues, are more challenging to manage and often lead to longer hospital stays. Additionally, the severity and outcome of a burn are influenced not just by its depth and size, but also by its location on the body. Burns in critical areas such as the face, hands, feet, and genitals, or those impacting the respiratory tract, are considered more severe and necessitate more complex treatments and intensive care. [5].

The epidemiology of burns varies significantly according to geographical and socio-economic context. In developed countries, most burns are caused by domestic accidents, while in developing countries, burns from open fires and occupational accidents are more common [6]. According to the World Health Organization (WHO) in 2008, burns were the fifth leading cause of mortality worldwide, with an estimated 180,000 people dying each year due to burns. In Spain, more than 1300 hospital admissions due to burns occur annually, and one in five people who arrive at emergency departments requires hospitalization for burns [7,8]. The epidemiological profile shows that children under 5 years of age and adults over 65 years of age are the most vulnerable groups, with a higher incidence of burns in the domestic environment [9]. Severe burns, involving more than 20% of the total body surface area, have a high morbidity and mortality rate, especially if they affect critical areas of the body such as the face, hands, feet, and perineum [10,11].

The initial management of burns in the emergency setting is critical to improve long-term outcomes. This includes a rapid assessment of the airway, breathing and circulation, known as the ABC (Airway, Breathing, Circulation) assessment, as well as pain control, infection prevention [12,13], stabilization of the patient, assessing the need for transfers to specialized burn units, and administration of analgesia, antibiotics, prophylactics and local wound care such as debridement and application of specific dressings [14]. Fluid resuscitation is essential in extensive burn patients to prevent hypovolemic shock, and the use of specific formulas such as the Parkland formula to calculate fluid requirements in the first 24 h is recommended [15]. Pain management is another priority, as burns are notoriously painful and may require a combination of opioid and non-opioid analgesics [16].

Nurses play a crucial role in burn management, assessing severity and providing immediate care. They adapt care according to age, skin thickness, and burn degree, participate in medical and surgical treatments, manage pain, and prevent infections. Additionally, they provide emotional support for long-term recovery and use study data to improve practices and ongoing education. Their role is vital for the comprehensive treatment and recovery of burn patients.

The duration of hospital admission varies significantly depending on the severity of the burn and response to treatment, ranging from a few days in mild cases to several weeks or months in severe cases [17]. Likewise, emergency care of burn patients should also consider psychological management, as these injuries often have a considerable emotional impact, both in the short and long term. Early interventions and a multidisciplinary approach can significantly improve patients’ long-term outcomes and quality of life [18].

Nurses intervene in multiple procedures for burn patients. They clean and disinfect wounds, apply and change dressings, and manage drainage devices. They administer transfusions, provide nutritional supplements, and insert intravenous lines. They offer emotional and physical support, helping with pain and stress management. They assist in skin replacement and excision, tissue repair, and removal of dressings and sutures. They support amputations and rehabilitation, facilitate tissue transfers, and help release contractures, ensuring the patient’s mobility and functionality.

The present investigation aims to describe the epidemiological–clinical characteristics of the burns attended (biological gender, age, length of stay in days, paediatric or adult emergency, main diagnostic type according to ICD-10 [19], skin thickness, degree of burn, body areas, % body surface area, and type of treatment received) in the Hospital Emergency Department of the Hospital Complex of Cáceres (Spain), which is composed of the University Hospital of Cáceres and the Hospital San Pedro de Alcántara, from January 2018 to December 2022; and to explore the differences in treatment according to biological gender, age, skin thickness affected, and degree of burn.

## 2. Materials and Methods

### 2.1. Participants

The patients participating in the study were those attended by the Emergency Hospital Service of the Cáceres Hospital Complex (Spain) between January 2018 and December 2022 due to some type of burn. Since the data needed to develop this study were requested from the Coding Service of the Hospital Complex of Cáceres, it is understood that it is not necessary to have information on the patient’s clinical history, since the data were handled anonymously. Therefore, we only worked with the number of daily burns and this would not be in breach of the Organic Law on Data Protection. In view of the above, neither the design nor the need for the patient to sign “Informed consent to carry out this study” was necessary. However, the approval of the Research Ethics Committee of the Cáceres Hospital Complex was necessary, and was favourable in the minutes n° 2 of 23 February 2023, with reference Code CEIm.-104-2022.

As inclusion criteria, the main diagnosis of the patients had to be coded according to the ICD-10 Classification as: T20–T25 Burns and corrosions of the external surface of the body, specified by location; T26–T28 Burns and corrosions limited to eyes and internal organs; or T30–T32 Burns and corrosions of multiple body regions and unspecified location [19]. All patients with second- or third-degree burns affecting more than 20% of body surface area, with second- or third-degree burns affecting more than 10% of body surface area in those over 50 years of age, with chemical and electrical burns, or with burns in critical areas (hands, feet, face and/or genitalia) are excluded, as they are not treated in this hospital emergency department but are referred to a specific burns unit. Also excluded were all those patients who, although they had been attended by the out-of-hospital emergency services due to a burn, were not admitted to the hospital emergency department for any reason.

From the total number of patients treated in the emergency department during that period (605,084 patients) 220 of them, 0.036%, were included in this research as they met the established criteria.

The ethical principles set out in the Declaration of Helsinki promulgated by the World Medical Association in 1964 were followed for this research.

### 2.2. Procedure

A retrospective, observational study was carried out on the incidence of burns cases attended in the years 2018–2022, following the STROBE recommendations for observational studies [20]. Data from patients who met the inclusion criteria were obtained from the medical records of the San Pedro de Alcántara hospital where all the data required for this study are routinely collected.

The data were collected by the hospital emergency staff, always health care personnel who provided care to the patients, which was collected in the clinical history. After discharge, the coding service coded the entire clinical process carried out on the patient with the ICD-10 codes, creating an appropriate database to perform the necessary statistical analyses.

The variables collected were discharge service, biological gender (male, female), age (years), date of admission, length of stay (days), type of emergency (paediatric or adult), classification according to the All Patients Refined—Diagnosis-Related Groups (APR-GRD) State Standard [21,22], type of treatment (medical/surgical) [19], main diagnosis according to ICD-10 (T20, T21…, T32), skin thickness affected (partial, complete, not stated), body surface area, degree of burn (first, second, third, not stated), areas affected (1, 2 or more, not stated), body surface area (partial, complete, not stated, body surface area not stated), number of burns (partial, complete, not stated, body surface area not stated); (T32), thickness of skin affected (partial, complete, not stated), degree of burn (first, second, third, not stated), areas affected (1, 2 or more, not stated), body surface area (according to ICD-10 classification), treatment approach (external, open, both, not stated), number of procedures required, type of procedure performed (replacement, excision, repair, removal, amputation, transfer, release, wound care, irrigation, dressing, drainage, transfusion, supplementation, insertion, insertion, support). Nursing actively participates in all these procedures, playing a pivotal role in each of them.

### 2.3. Statistical Analysis

Statistical analysis was performed with IBM-SPSS V.25.0 software.

A descriptive analysis was performed for all variables, so that for quantitative variables the mean and standard deviation (SD) were calculated, and for categorical variables the distribution of frequencies and percentages was calculated.

To analyse the existence of differences in treatment between the different groups according to biological gender, age, thickness of skin affected and degree of burn, Student’s *t*-statistics, Chi-square or Anova were used according to each case; a *p*-value < 0.05 was considered statistically significant. Those patients in which the thickness of the skin affected or the degree of the burn was not recorded were excluded from the analyses in which these variables were involved.

## 3. Results

This section may be divided by subheadings. It should provide a concise and precise description of the experimental results and their interpretation, as well as the experimental conclusions that can be drawn.

### 3.1. Descriptive Analysis

Analyses were performed on 220 patients; 62 of them seen in 2018 (28.2%), 42 in 2019 (19.1%), 28 in 2020 (12.7%), 53 in 2021 (24.1), and 35 in 2022 (15.9%).

The main diagnosis coded according to the ICD-10 classification is distributed in 210 persons (95.5%) in the group T20–T25 Burns and corrosions of the external surface of the body, specified by location; 1 person (0.5%) in the group T26–T28 Burns and corrosions limited to eye and internal organs; and 4 persons (1.8%) in the group T30–T32 Burns and corrosions of multiple body regions and unspecified location.

The remaining descriptive statistics are shown in Table 1 and Table 2.

### 3.2. Differences in Treatment Procedures According to Biological Gender and Age

There are no differences in the type of treatment procedure(s) used according to biological gender in any case.

Table 3 shows the significant differences in the treatment procedures used or not according to age. For all other procedures (repair, removal, amputation, transfer, irrigation, dressing, drainage, transfusion, supplementation, insertion, insertion, and support) there are no significant differences. The Anova also shows no relationship between age and type of approach (external, open, both) (F(2, 156) = 1.093, *p* = 0.338).

On the other hand, age correlates with the number of procedures performed (r(220) = 0.150, *p* = 0.026) and with the number of days of hospital stay (r(220) = 0.181, *p* = 0.007), so that the older the age, the more procedures performed and the more days of hospital stay required.

### 3.3. Differences in Treatment Procedures According to Affected Skin Thickness and Degree of Burn

Table 4 shows the significant differences in the treatment procedures used according to the thickness of the affected skin and the degree of the burn; the expected counts and counts are available in Supplementary Material S1.

There are also no differences in the number of days of admission or the number of procedures performed according to the thickness of skin affected or the degree of the burn.

## 4. Discussion

The aim of this study was to describe the epidemiological–clinical characteristics of burns treated in the emergency department and, in addition, to explore the differences in treatment according to biological sex, age, thickness of the affected skin, and degree of burn.

Our results have allowed us to delineate a specific profile of the epidemiological–clinical characteristics of the burns treated in the Emergency Department of the Hospital Complex of Cáceres between January 2018 and December 2022. During this period, 220 patients with a mean age of 47 years were analysed, with a predominance of men (60.9%). Most patients (84.5%) were adults and 95.5% had burns and corrosions on the external surface of the body (ICD-10 classification T20–T25). These patients had a mean hospital stay of 7.86 days and received a mean of 2.7 treatment procedures.

In terms of treatment, 75.5% of patients received medical treatment, while 24.5% required surgical intervention. According to the APR_GRD classification, 63.2% of patients had partial-thickness burns, followed by those with full-thickness burns. Most of the burns affected less than 10% of the body surface. 63.3% of the patients had burns partially affecting the thickness of the skin, while 27.7% had full-thickness burns. As for the degree of burns, 46.8% were second degree, 21.8% were third degree and the rest were first degree, although the degree was not specified in 21.8% of cases. The ED approach was mostly external (30.5%), followed by a combined external-open (30%) and open (11.8%) approach.

Our findings are consistent with previous studies reporting a higher prevalence of burns in adult men. For example, a study by Rodriguez-Salazar in a hospital in Cuba also found that 63% of burn patients were male, with a mean age of 39 years, similar to our sample [23]. In terms of location and type of burn, another Mexican study found that most of the burns in their study were superficial and affected less than 55% of the body surface [24]. In our study, 63.3% of patients had burns affecting less than 10% of the body surface, suggesting a similar trend in the severity and extent of burns.

The mean hospital stay of 7.86 days in our study compares with another study whose mean was 15 days for minor injuries and 19.4 days for severe injuries [25]. However, our mean of 2.7 treatment procedures per patient is lower compared to the findings of Demirdjian and Muñoz, who reported a mean of 4.5 procedures per patient [26]. This could be due to differences in treatment protocols or in the severity of burns treated. Regarding the type of treatment, our finding is consistent with trends observed in research where they found that most burns can be managed without surgical intervention [27].

On the other hand, in terms of the second objective of the study, it can be observed in our results that no significant differences were obtained in terms of the biological sex of the affected persons in any case. Nor was a relationship found between age and the type of approach that was carried out, i.e., external, open or both. However, statistically significant differences can be observed in the treatment procedures used as a function of age. Treatment procedures by substitution, excision, release, and VDA/wound care can be highlighted. Other studies agree with our findings regarding the lack of significant differences between biological sex and type of burn treatment [28,29]. This suggests that treatment decisions are primarily based on the severity and extent of burns, rather than the sex of the patient.

Regarding the relationship between age and treatment procedures, our results show a trend that differs from some previous studies. For example, one investigation reported that older patients tended to receive less invasive treatments due to comorbidities and elevated surgical risks [30]. However, our study found that substitution and excisional procedures were more common in older patients, which could indicate a greater willingness or need for more aggressive surgical interventions in this population in our specific context. Regarding release and DSA/wound-care procedures, our results are congruent with those of Adams and Sheridan who found that younger patients tended to receive treatments focused on release and wound care due to their resilience and lower risk of complications [31].

On the other hand, we found a correlation with the number of procedures performed and the length of hospital stay, so that it can be observed that the older the patient, the more procedures are performed and the longer the length of hospital stay. Our findings are consistent with the results of previous studies suggesting a relationship between older age, the number of medical procedures performed, and length of hospital stay. A similar correlation is found in a study of burn patients, where older patients required more medical interventions and had longer hospital stays [32]. Furthermore, a study by Kottner also reported that older patients often have more severe injuries and secondary complications that require additional interventions and prolong hospital stay [33]. On the other hand, research corroborates that older patients, due to frailty and decreased resilience, not only require more interventions, but also experience slower recovery, which prolongs their hospitalization time [34].

Significant differences can also be observed in our results with the treatment procedures that were employed with respect to the thickness of the skin that was affected by the burn, and the degree of the burn. The excision procedure was performed more often than expected in the case of complete involvement of the skin thickness, as was the case with the transfer procedure. Also, taking into account the degree of the burn, the excision procedure was performed more frequently in second-degree burns; however, it was performed more often than expected in third-degree burns. The amputation procedure was only performed on third-degree burns in a higher than expected manner (according to Chi-square results), as was the transfer procedure. Finally, the ADL/wound-care procedure was performed in second-degree burns more than expected (according to Chi-square results), and with a significant percentage compared to first- and third-degree burns. However, no differences were observed according to the days of admission and the number of procedures carried out taking into account the thickness of the skin affected and the degree of the burn.

Regarding the frequency of excisional procedures in second-degree burns, our findings are compatible with a study noting that second-degree burns often require excision to accelerate healing and reduce the risk of hypertrophic scarring [35]. However, our finding that excisions were also performed more frequently in third-degree burns suggests an adaptation to the severity of the injuries to avoid severe complications, which is also supported by the findings of [36].

Regarding the use of the amputation procedure exclusively in third-degree burns, our results are consistent with the study that documented that amputations are often unavoidable in third-degree burns that compromise limb viability due to complete tissue destruction [37]. The frequency of DSA/wound-care procedures in second-degree burns is also supported by other research indicating that these procedures are crucial for maintaining adequate moisture and preventing infection, which is essential for optimal recovery in second-degree burns [38].

This research study has several strengths that contribute to its value. It provides valuable insights into the epidemiology and outcomes of burn patients in a specific region, offering a focused analysis that can guide local healthcare improvements. The inclusion of 220 patients provides a substantial dataset for examining patterns and trends in burn injuries. The study’s retrospective design allows for the analysis of real-world data over an extended period, which can reveal long-term trends and outcomes. Additionally, the use of comprehensive medical records ensures that a wide range of patient variables and treatment details are considered. The study also highlights the importance of specialized burn care and the role of dedicated burn units in improving patient outcomes. By identifying common causes and characteristics of burns, the study can inform prevention strategies and public health initiatives. Finally, the research contributes to the limited body of literature on burn injuries in the specific geographical context of Cáceres, Spain, adding valuable data for comparison with other regions and enhancing the overall understanding of burn-injury epidemiology.

This research in the field of burns strengthens nursing practice by providing solid evidence that supports informed clinical decisions and improves outcomes for patients affected by these injuries. Nursing plays a vital role in severity assessment, immediate care, and emotional support. Nurses participate in medical and surgical management, pain control, infection prevention, and ongoing education. This study analyses epidemiological and clinical data of burn patients, highlighting demographic features, burn types, and treatments, essential knowledge for tailoring patient care.

The study has several limitations to consider. Firstly, its retrospective design limits the ability to establish causality and may introduce biases due to the accuracy of medical records. Since this was a retrospective study, only the data available at the hospital up to the time of the study could be used, which meant that some potentially important variables could not be included. This limitation will be addressed in future research, where additional variables such as the causes of burns, treatment with hyperbaric oxygen therapy, and discharge management, among others, can be incorporated. The small sample size of 220 patients may not be representative of the broader population, and the lack of detailed data on burn severity, skin thickness, and burn locations affects the accuracy of the analysis. Additionally, the findings are based on a single hospital in Cáceres, Spain, and may not be applicable to other regions or socioeconomic contexts. The results also do not apply to patients with severe burns affecting more than 20% of the body or critical areas. It is also worth mentioning that it has not been possible to collect data on major burns, which will be taken into account in future studies as the research continues. Furthermore, differences in treatment approaches and documentation among healthcare providers may introduce variability. The study does not evaluate the psychological effects on burn patients, an important aspect of long-term care. Finally, there is limited analysis of potential confounding factors such as comorbidities and treatment variations. These limitations highlight the need for future prospective and multicentre studies to validate and expand upon these findings.

## 5. Conclusions

In conclusion, this study provides a comprehensive analysis of the epidemiological and clinical characteristics of burns treated in the Emergency Department of the Hospital Complex of Cáceres between 2018 and 2022. The findings delineate a specific profile of burn patients, predominantly adult men with burns primarily affecting the external body surface. Treatment predominantly involved medical interventions rather than surgical intervention, with significant differences observed based on age, and thickness and degree of burns. These insights contribute valuable data to the field of burn injury management and underscore the importance of personalized approaches to improve patient outcomes. Additionally, they highlight the crucial role of nursing in severity assessment, immediate care, and emotional support, as well as in medical and surgical management, pain control, infection prevention, and ongoing education.

## Figures and Tables

**Table 1:** Descriptive statistics of quantitative variables.

	N	Minimum	Maximum	Mean	Standard Deviation
Age (years)	220	0	90	47.04	25.64
Hospital stay (days)	220	0	46	7.86	7.09
Number of treatment procedures	220	0	8	2.7	2.09

**Table 2:** Frequencies and percentages of qualitative variables.

		Frecuency	%
Biological gender	Male	134	60.9
	Female	86	39.1
Type of emergency	Paediatric	34	15.5
	Adult	186	84.5
Type of treatment	Medical	166	75.5
	Surgical	54	24.5
APR_GRD Classification	361—Skin grafting for skin and subcutaneous tissue diagnoses	4	1.8
	364—Other skin, subcutaneous tissue and related procedures	4	1.8
	815—Other diagnostic injury, poisoning and toxic effects	0	0.0
	816—Toxic effects of non-medicinal substances	2	0.9
	842—Full-thickness burns with skin grafting	42	19.1
	843—Extensive third-degree or full-thickness burns without skin grafting	19	8.6
	844—Partial-thickness burns with or without skin grafting	139	63.2
	850—Procedure with diagnosis of rehabilitation, aftercare or other contact with health services	4	1.8
	862—Other aftercare and convalescence	3	1.4
	951—Moderately extensive procedure not related to principal diagnosis	1	0.5
	Lost	2	0.9
Body surface area affected	T31.0—Burns with less than 10% body surface area involvement. T31.10—Burns with 10–19% body surface area involvement with 0–9% of third-degree burns	80	36.4
	T31.10—Burns involving 10–19% of body surface area with 0–9% of third-degree burns	8	3.6
	T31.11—Burns involving 10–19% of body surface area with 10–19% of third-degree burns. T31.20—Burns involving 10–19% of body surface area with 10–19% of third-degree burns	1	0.5
	T31.20—Burns involving 20–29% of body surface area with 0–9% of third-degree burns	1	0.5
	T31.30—Burns involving 30–39% of body surface area with 0–9% of third-degree burns	1	0.5
	T32.0—Corrosion involving less than 10% of body surface area	1	0.5
	T32.10—Corrosions affecting 10–19% of body surface area with 0–9% third-degree corrosion	1	0.5
	Not stated	127	57.7
Thickness of skin affected	Partial	139	63.2
	Complete	61	27.7
	Not stated	20	9.1
Degree of burn	First	3	1.4
	Second	203	46.8
	Third	66	30.0
	Not stated	48	21.8
Approach	External	67	30.5
	Open	26	11.8
	Both	66	30.0
	Not stated	61	27.7

**Table 3:** Differences in treatment procedures used or not according to age. Student’s *t*-test.

Procedures		N	Age Mean	Age SD	t	*p*-Value
Substitution	Yes	117	53.03	23.77	3.80	<0.001
	No	103	40.24	26.10		
Excision	Yes	133	51.93	23.55	3.51	<0.001
	No	87	39.72	27.08		
Release	Yes	2	11.50	12.02	−1.98	0.024
	No	218	47.37	25.51		
ADL/Wound care	Yes	19	33.53	26.24	−2.43	0.008
	No	201	48.32	25.28		

**Table 4:** Differences in the treatment procedures used according to the thickness of skin affected and the degree of burn. Chi-square.

		Value	Bilateral Exact Significance (Fisher’s Exact Test)	Bilateral Asymptotic Significance
Thickness	Excision	5.16	0.027	
	Transfer	9.30	0.008	
Degree	Excision	6.21		0.045
	Amputation	6.58		0.037
	Transfer	6.58		0.037
	ADL/Wound care	6.92		0.031

## Data Availability

Data supporting reported results can be found at the institutional repository of the University of Burgos: http://hdl.handle.net/10259/9358.

## Supplementary Materials

The following supporting information can be downloaded at: https://www.mdpi.com/article/10.3390/nursrep14030148/s1, Supplementary Material S1: Differences in treatment procedures used depending on the thickness of skin affected and the degree of burn. Cross tabulations with counts and expected counts.

## Public Involvement Statement

No public involvement in any aspect of this research.

## Guidelines and Standards Statement

This manuscript was drafted against the required Nursing Reports template. Cites and reference were as recommended by the ACS style guide.
