# Supplementary material for: Retrospective Study of the Epidemiological–Clinical Characteristics of Burns Treated in a Hospital Emergency Service (2018–2022)
Source: Nurs Rep. 2024 Aug 14;14(3):1987–97. doi: 10.3390/nursrep14030148 (PMC11348191; doi:10.3390/nursrep14030148)
Supplement: Supplementary file 1 [file nursrep-14-00148-s001.zip › nursrep-3109654-supplementary.pdf]

Supplementary Material S1

Differences in treatment procedures used depending on the thickness of skin affected and the degree of burn. Cross tabulations with counts and expected counts.

THICKNESS (partial/complete) - EXCISION PROCEDURE (yes/no)

			Excision Procedure		
			YES	NO	Total
**THICKNESS of skin affected**	PAR TIAL	Count	79	60	139
		Expected recount	86.2	52.8	139.0
		% within THICKNESS	56.8%	43.2%	100.0%
	CO MPL ETE	Count	45	16	61
		Expected recount	37.8	23.2	61.0
		% within THICKNESS	73.8%	26.2%	100.0%
**Total**		Count	124	76	200
		Expected recount	124.0	76.0	200.0
		% within THICKNESS	62.0%	38.0%	100.0%

THICKNESS (partial/complete) - TRANSFER PROCEDURE (yes/no)

			Transfer Procedure		
			YES	NO	Total
**THICKNESS of skin affected**	YES	Count	0	139	139
		Expected recount	2.8	136.2	139.0
		% within THICKNESS	0.0%	100.0%	100.0%
	NO	Count	4	57	61
		Expected recount	1.2	59.8	61.0
		% within THICKNESS	6.6%	93.4%	100.0%
**Total**		Count	4	196	200
		Expected recount	4.0	196.0	200.0
		% within THICKNESS	2.0%	98.0%	100.0%

DEGREE OF BURN (first, second, third) - EXCISION PROCEDURE (yes/no)

			Excision Procedure		
			**YES**	**NO**	**Total**
**DEGREE of burn**	First	Count	1	2	3
		Expected recount	1.8	1.2	3.0
		% within DEGREE	33.3%	66.7%	100.0&
	Second	Count	55	48	103
		Expected recount	61.7	41.3	103.0
		% within DEGREE	53.4%	46.6%	100.0%
	Third	Count	47	19	66
		Expected recount	39.5	26.5	66.0
		% within DEGREE	71.2%	28.8%	100.0%
**Total**		Count	103	69	172
		Expected recount	103.0	69.0	172.0
		% within DEGREE	59.9%	40.1%	100.0%

DEGREE OF BURN (first, second, third) - AMPUTATION PROCEDURE (yes/no)

			Amputation Procedure		
			**YES**	**NO**	**Total**
**DEGREE of burn**	First	Count	0	3	3
		Expected recount	.1	2.9	3.0
		% within DEGREE	0.0%	100.0%	100.0%
	Second	Count	0	103	103
		Expected recount	2.4	100.6	103.0
		% within DEGREE	0.0%	100.0%	100.0%
	Third	Count	4	62	66
		Expected recount	1.5	64.5	66.0
		% within DEGREE	6.1%	93.9%	100.0%
**Total**		Count	4	168	172
		Expected recount	4.0	168.0	172.0
		% within DEGREE	2.3%	97.7%	100.0%

DEGREE OF BURN (first, second, third) - TRANSFER PROCEDURE (yes/no)

			Transfer Procedure		
			YES	NO	Total
**DEGREE of burn**	First	Count	0	3	3
		Expected recount	.1	2.9	3.0
		% within DEGREE	0.0%	100.0%	100.0%
	Second	Count	0	103	103
		Expected recount	2.4	100.6	103.0
		% within DEGREE	0.0%	100.0%	100.0%
	Third	Count	4	62	66
		Expected recount	1.5	64.5	66.0
		% within DEGREE	6.1%	93.9%	100.0%
**Total**		Count	4	168	172
		Expected recount	4.0	168.0	172.0
		% within DEGREE	2.3%	97.7%	100.0%

DEGREE OF BURN (first, second, third) – WOUND CARE PROCEDURE (yes/no)

			Wound Care Procedure		
			YES	NO	Total
**DEGREE of burn**	First	Count	1	2	3
		Expected recount	.3	2.7	3.0
		% within DEGREE	33.3%	66.7%	100.0%
	Second	Count	14	89	103
		Expected recount	10.2	92.8	103.0
		% within DEGREE	13.6%	86.4%	100.0%
	Third	Count	2	64	66
		Expected recount	6.5	59.5	66.0
		% within DEGREE	3.0%	97.0%	100.0%
**Total**		Count	17	155	175
		Expected recount	17.0	155.0	172.0
		% within DEGREE	9.9%	90.1%	100.0%
